# Effects of Levofloxacin, Aztreonam, and Colistin on Enzyme Synthesis by *P. aeruginosa* Isolated from Cystic Fibrosis Patients

**DOI:** 10.3390/antibiotics11081114

**Published:** 2022-08-17

**Authors:** Arianna Pani, Valeria Lucini, Silvana Dugnani, Alice Schianchi, Francesco Scaglione

**Affiliations:** Oncology and Hemato-Oncology, University of Milan, 20122 Milan, Italy

**Keywords:** cystic fibrosis, *P. aeruginosa*, antibiotics

## Abstract

(1) Background: Cystic fibrosis (CF) is characterized by chronic pulmonary inflammation and persistent bacterial infections. *P. aeruginosa* is among the main opportunistic pathogens causing infections in CF. *P. aeruginosa* is able to form a biofilm, decreasing antibiotic permeability. LOX, a lipoxygenase enzyme, is a virulence factor produced by *P. aeruginosa* and promotes its persistence in lung tissues. The aim of this study is to evaluate if antibiotics currently used for aerosol therapy in CF are able to interfere with the production of lipoxygenase from open isolates of *P. Aeruginosa* from patients with CF. (2) Methods: Clinical isolates of *P. aeruginosa* from patients with CF were grown in Luria broth (LB). Minimum inhibitory concentration (MIC) was performed and interpreted for all isolated strains according to the European Committee on Antimicrobial Susceptibility Testing (EUCAST) guidelines. We selected four antibiotics with different mechanisms of action: aztreonam, colistin, amikacin, and levofloxacin. We used human pulmonary epithelial NCI-H929 cells to evaluate LOX activity and its metabolites according to antibiotic action at increasing concentrations. (3) Results: there is a correlation between LOX secretion by clinical isolates of *P. aeruginosa* and biofilm production. Levofloxacin exhibits highly significant inhibitory activity compared to the control. Amikacin also exhibits significant inhibitory activity against LOX production. Aztreonam and colistin do not show inhibitory activity. These results are also confirmed for LOX metabolites. (4) Conclusions: among the evaluated antibiotics, levofloxacin and amikacin have an activity on LOX secretion.

## 1. Introduction

Bacterial infection and linked chronic pulmonary inflammation are a pathological trait associated with the genetic disease cystic fibrosis (CF) [1]. These bacterial infections are characterized by robust inflammatory responses, with an elevation in the levels of proinflammatory cytokines and neutrophil accumulation in the CF airway. Unfortunately, responses triggered by the inflammation are not effective in clearing pathogenic microbes in the CF lung [2], creating instead a hyperinflammatory status which can lead to the damage of host tissues and respiratory failure, leading to transplantation or death [3].

Most adult patients with CF have a chronic infection of the airways caused by the opportunistic bacterial pathogen *P. aeruginosa*, which is frequently associated with morbidity and mortality. *P. aeruginosa* grows in the context of hyperinflammation in CF lungs and is able to form mechanically robust biofilms which are resistant to clinically achievable levels of antibiotics [4].

Antibiotic classes currently approved in many countries for use by inhalation include the aminoglycosides (tobramycin and amikacin), monobactams (aztreonam), polymyxins (colistimethate) and fluoroquinolones (levofloxacin). Although all of the antibiotics used are beneficial, none of them are capable of completely eradicating *P. aeruginosa* from bronchial secretions in CF. However, relatively recent studies have reported that levofloxacin, administered by aerosol in CF, produces positive effects that go beyond its simple antibacterial effect [5,6,7].

*P. aeruginosa* also persists in the airways by interfering with host defense via secreted bacterial virulence factors and small molecules. Recently, it has been demonstrated that several *P. aeruginosa* clinical isolates express *LoxA*, a gene which encodes for the lipoxygenase enzyme (LOX) that oxidizes polyunsaturated fatty acids [8]. In the lungs, LOX is able to process a wide range of host polyunsaturated fatty acids, with a consequent production of bioactive lipid mediators (including lipoxin A4). LOX is also able to inhibit major chemokine expression, such as macrophage inflammatory proteins (MIPs) and keratinocytes-derived chemokines (KCs), and to recruit leukocytes. Importantly, LOX is able to promote *P. aeruginosa* survival in lung tissues, suggesting a LOX-dependent interference between the host lipid pathways and *P. aeruginosa* lung pathogenesis.

In this study, we wanted to verify whether the antibiotics currently used via aerosols in CF could interfere with the production of LOX from isolates of *P. aeruginosa* from patients with CF.

## 2. Results

### 2.1. Antibiotic Intrinsic Activity (MICs)

In order to evaluate the activity of antibiotics approved for aerosol use in cystic fibrosis on the production of LOX by *P. aeruginosa*, we chose four antibiotics representing different mechanisms of action: levofloxacin, amikacin, aztreonam, and colistin. First, we evaluated the antibiotic intrinsic activity against 12 clinically isolated strains of *P. aeruginosa*.

Table 1 shows the minimum inhibitory concentrations (MICs) of the levofloxacin, amikacin, aztreonam, and colistin studied against the clinically collected strains of *P. aeruginosa*. Strains 1, 2, 3, 9, 10, 11, and 12 were resistant to levofloxacin (MIC > 2). Only strain 12 was resistant to amikacin (MIC > 16) and strains 9 and 10 to aztreonam (MIC > 16). Strains 1, 2, and 11 were resistant to colistin (MIC > 4). Low MICs (≤0.25 mg/L) were detected for levofloxacin, amikacin, aztreonam, and colistin in strains 4 and 8.

### 2.2. Biofilm Formation

Secondly, we evaluated the ability of the 12 clinically isolated strains to produce biofilm.

**Figure 1:**
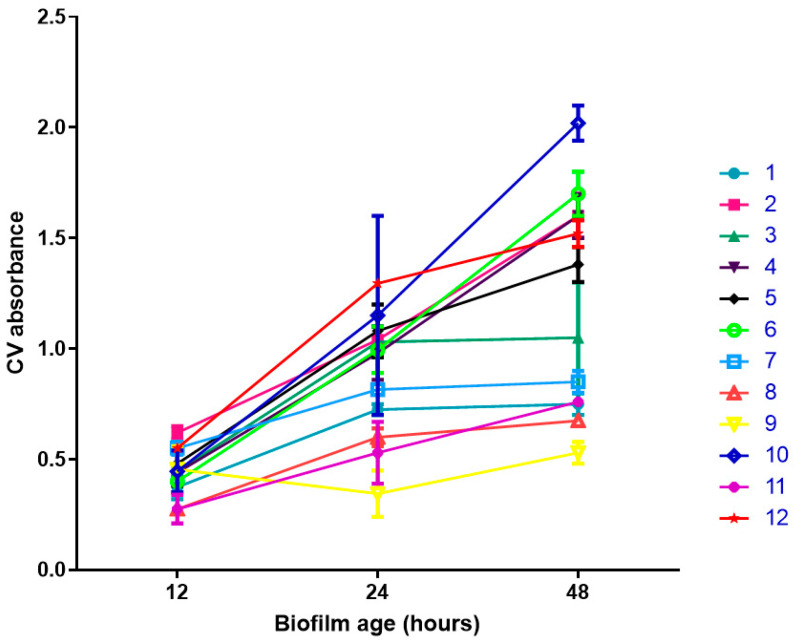
**Ability to form** biofilm for the 12 clinically isolated *P. aeruginosa* strains. Biofilm was evaluated at 12, 24, and 48 h. CV absorbance measures biofilm formation. Each colored line (numbers from 1 to 12, see Table 1) refers to a different *P. aeruginosa* strain.

Figure 1 shows the biofilm formation by the 12 selected strains. As expected, there are different abilities to form biofilms. Strains 2, 4, 6, and 10 demonstrated the highest ability to produce biofilms at 12, 24, and 48 h. Strain 2 showed a CV absorbance of 0.620 (0.042) at 12 h, 1.040 (0.085) at 24 h, and 1.600 (0.141) at 48 h. Strain 4 showed a CV absorbance of 0.440 (0.127) at 12 h, 0.980 (0.170) at 24 h, and 1.600 (0.141) at 48 h. Strain 6 showed a CV absorbance of 0.400 (0.014) at 12 h, 0.995 (0.148) at 24 h, and 1.700 (0.141) at 48 h. Strain 10 showed a CV absorbance of 0.445 (0.134) at 12 h, 1.150 (0.636) at 24 h, and 2.020 (0.113) at 48 h.

### 2.3. LOX Activity

Since the expression of the loxA gene differs from one *P. aeruginosa* strain to another, especially in biofilm growth conditions [9], we measured the LOX activity of the 12 clinically isolated strains. The aim of our evaluations of biofilm and LOX activity was to select the best strain on which to continue our evaluation on the impact of antibiotics on LOX production.

**Figure 2:**
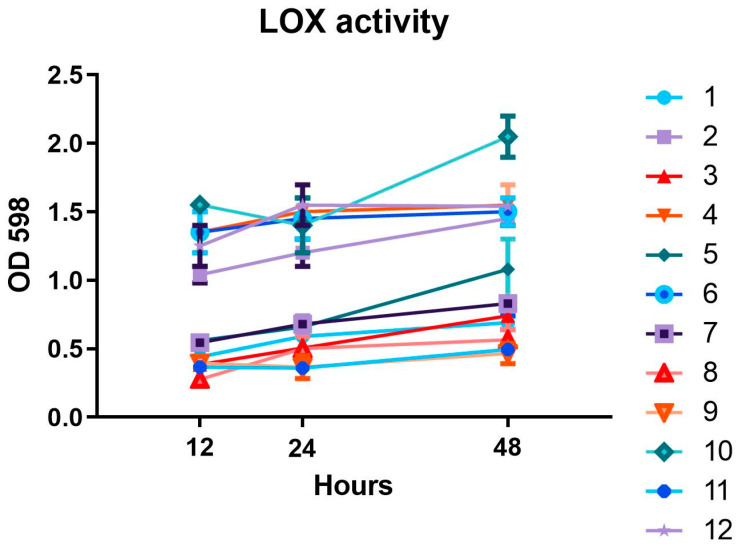
Lipoxygenase (LOX) activity of the 12 selected *P. aeruginosa* strains. LOX activity was evaluated at 12, 24, and 48 h. Each colored line (numbers from 1 to 12, see Table 1) refers to a different *P. aeruginosa* strain.

Figure 2 shows the LOX activity of the 12 selected strains. From the comparison of Figure 1 and Figure 2 it emerges that the strains with the greatest ability to make biofilms are those that have the greatest LOX activity. By performing a one-way ANOVA with Bonferroni’s correction, the LOX activity differs statistically significantly between the 12 strains (*p* < 0.001). In fact, strains 2, 4, 6, and 10 demonstrated the highest LOX activity at 12, 24, and 48 h. Mean absorbance of strain 2 was 1.040 (0.085) at 12 h, 1.200 (0.141) at 24 h, and 1.450 (0.071) at 48 h. Mean absorbance of strain 4 was 1.350 (0.212) at 12 h, 1.500 (0.141) at 24 h, and 1.550 (0.212) at 48 h. Mean absorbance of strain 6 was 1.350 (0.212) at 12 h, 1.450 (0.212) at 24 h, and 1.500 (0.141) at 48 h. Mean absorbance of strain 10 was 1.550 (0.071) at 12 h, 1.400 (0.283) at 24 h, and 2.050 (0.212) at 48 h.

Considering these results, the number 10 strain of *P. aeruginosa* was chosen to perform the subsequent experiments.

### 2.4. Effects of Antibiotics on LOX Activity

**Figure 3:**
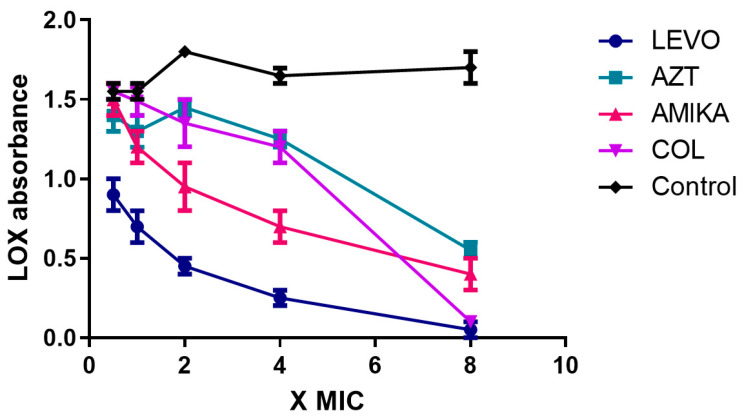
Effects of levofloxacin, aztreonam, amikacin, and colistin on the LOX activity of a clinically isolated strain of *P. aeruginosa* with high LOX activity. Antibiotics were tested at increasing concentrations (0.5- to 8-fold the MICs). (LEVO = levofloxacin, AZT = aztreonam, AMIKA = amikacin, COL = colistin, Control = untreated strain).

We then evaluated the effect of the levofloxacin, aztreonam, amikacin, and colistin on the LOX activity of strain 10 of *P. aeruginosa*. Results are shown in Figure 3.

Levofloxacin exhibits highly significant inhibitory activity compared to the control (mean absorbance at four times the MIC—0.250 (0.071) vs. 1.650 (0.071)). To a lesser extent, amikacin also exhibits significant inhibitory activity (mean absorbance at four times the MIC—0.700 (0.141) vs. 1.650 (0.071)), while aztreonam and colistin do not show inhibitory activity (mean absorbance at four times the MIC, respectively 1.250 (0.071) and 1.200 (0.141)). It should be noted that at eight times the MIC all antibiotics show inhibitory activity. This is most likely related to bactericidal activity on the strain rather than to the activity inhibiting LOX synthesis.

We compared the effects of the four different antibiotics on LOX activity. Table 2 reports the results of the analysis. Differences between levofloxacin and aztreonam, colistin and aztreonam, and colistin were statistically significant (Table 2).

### 2.5. Production of 15-LOX-Dependent Metabolites in Lung Epithelial Cells Infected by P. aeruginosa

**Figure 4:**
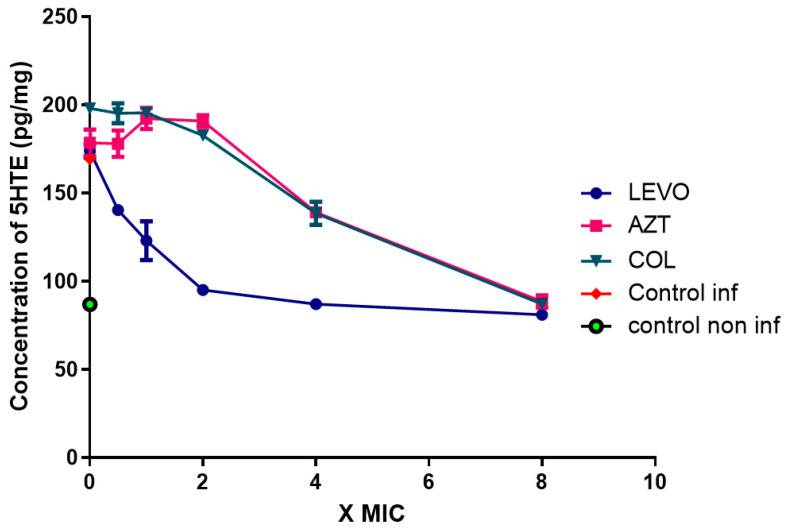
Concentration of 15-HETE (pg/mg of protein) in extracts of human lung epithelial NCI-H292 cells infected or noninfected with *P. aeruginosa*, 24 h post-infection. (LEVO = levofloxacin, AZT = aztreonam, COL = colistin, Control inf = control in infected cells, Control non inf = control in noninfected cells).

To evaluate LOX effects on the host response, we measured LOX metabolites in human lung epithelial NCI-H292 cells challenged by *P. aeruginosa* strain 10. We measured 15-Hydroxyeicosatetraenoic acid (15-HETE), 17-hydroxydocosahexaenoic acid (17-HDoHE) and lipoxin A4 production (LXA4). Figure 4 shows the results relating to the effect of the various antibiotics on the production of 15-HETE. Levofloxacin exhibits significant inhibitory activity compared to the control (mean difference −112.1, 95% CI 27.99–196.2, *p* < 0.005), while amikacin aztreonam, and colistin show no significant inhibitory activity. In this case it should also be noted that at eight times the MIC all antibiotics show inhibitory activity. This is most likely related to bactericidal activity on the strain rather than to the activity inhibiting LOX synthesis.

**Figure 5:**
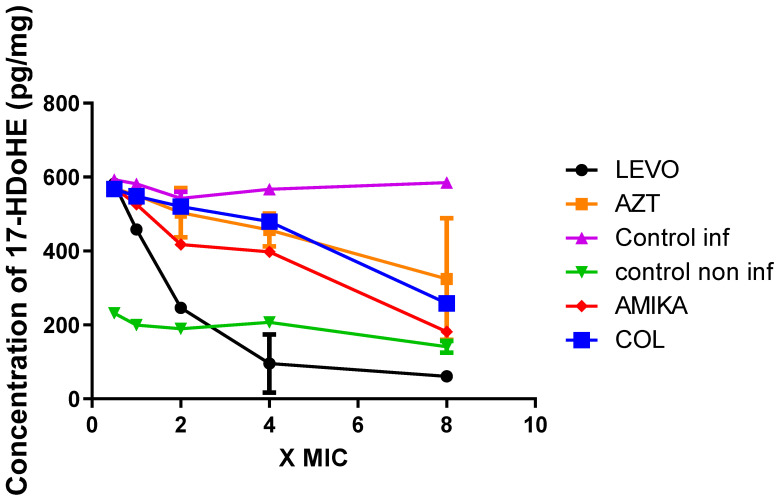
Concentration of 17-HDoHE (pg/mg of protein) in extracts of human lung epithelial NCI-H292 cells non-infected and treated with antibiotics, 24 h. (LEVO = levofloxacin, AZT = aztreonam, AMIKA = amikacin, COL = colistin, Control inf = control in infected cells, Control non-inf = control in noninfected cells).

Figure 5 shows the results relating to the effect of the various antibiotics on the production of 17-HDoHE. Only levofloxacin exhibits significant inhibitory activity compared to the control (mean difference −112 95% CI 55.4–514.8, *p* < 0.005), while amikacin, aztreonam, and colistin show no significant inhibitory activity. In this case it should also be noted that at eight times the MIC all antibiotics show inhibitory activity.

**Figure 6:**
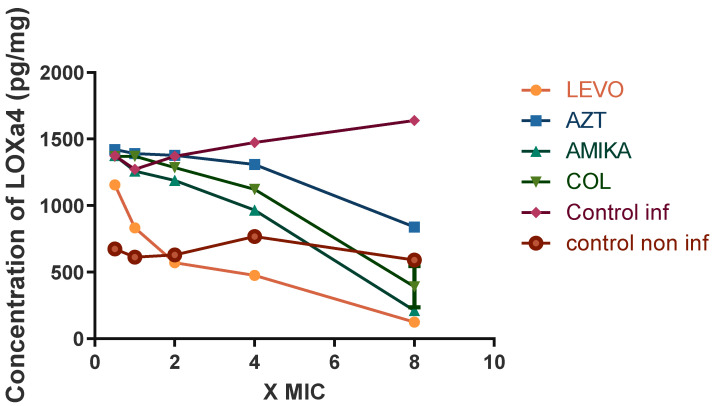
Concentration of LOXa4 (pg/mg of protein) in extracts of human lung epithelial NCI-H292 cells infected, noninfected, and treated with antibiotics after 24 h. (LEVO = levofloxacin, AZT = aztreonam, AMIKA = amikacin, COL = colistin, Control inf = control in infected cells, Control non inf = control in noninfected cells).

Figure 6 shows the results relating to the effect of various antibiotics on LXA44 production. Only Levofloxacin exhibits significant inhibitory activity compared to the control (mean difference −794.9, 95%CI 225–1364, *p* < 0.005). While amikacin aztreonam and colistin show no significant inhibitory activity. In this case it should also be noted that at eight times the MIC all antibiotics show inhibitory activity.

## 3. Discussion

*Pseudomonas aeruginosa* is a highly versatile bacterium. One basis for its versatility is the arsenal of enzymes that helps this pathogen to adapt to its environment. Our study shows that lipoxygenase (LOX) is secreted by clinical isolates of *P. aeruginosa*, producing biofilm, and this enzyme may contribute to the lung pathogenesis triggered by this opportunistic pathogen. In a series of elegant experiments, Morello et al. [8] have shown that LOX activity decreases release of chemokines such as KC (CXCL-1) and macrophage inflammatory proteins (MIP-1α/CCL-3, MIP-1β/CCL-4, and MIP-2/CXCL-2), producing a lower recruitment of immune cells in the airspaces. More importantly, they also observed that LOX activity promotes the spread of bacteria in lung tissues.

The role of 15-LOX has been implicated in various inflammation-related diseases. Increasing evidence highlights the controversial nature of 12/15-LOX in inflammation, as its metabolites have been shown to have both pro- and anti-inflammatory properties [10].

The pro-inflammatory role of 15-LOX and its metabolite 15(S)-HETE was demonstrated by various studies [11,12]. It is important to note that expression of pro-inflammatory cytokines IL-6, IL-12, CXCL9, and CXCL10, which is LPS-induced, is reduced by inhibition of 12/15-LOX in macrophages [13]. Furthermore, 15-LOX is able to regulate the expression of pro-inflammatory eoxins in epithelial airway cells and eosinophils. Eoxins can cause endothelial cell dysfunction and enhance vascular permeability [14]. Additionally, the increased expression of some of these inflammatory molecules is dependent on nuclear factor κB activation [15,16]. Moreover, recently it has been reported that 15-LOX may interact with secretory phospholipase A2 (sPLA2), contributing to sterile inflammation in chronic conditions with differences from classical inflammation on the cytokine level [17].

On the other hand, demonstrations of the anti-inflammatory properties of 12/15-LOX and its metabolites have been reported. The exact mechanisms by which 12/15-LOX explicates its anti-inflammatory effects are not fully understood, but they may be due to its pro-resolving mediators such as lipoxins, resolvins, and protectins, which are able to induce a potent and direct anti-inflammatory response in various cell types. In summary, it is becoming clear that 12/15-LOX and its metabolites have both pro- and anti-inflammatory effects, and some of these differential effects of the same metabolites could be due to their different concentrations [10].

In this study, we analyzed the activity of three antibiotics currently used in cystic fibrosis as antipseudomonal inhalation therapy. Among the antibiotics used via aerosol in CF, only levofloxacin exhibited significant inhibitory activity of LOX activity compared to the control, while aztreonam and colistin show no significant inhibitory activity. Levofloxacin acting on the synthesis of enzymes by *P. aeruginosa* determines an indirect anti-inflammatory effect, which is very useful in CF. These data may explain the positive effects exceeding those attributable to the antibacterial activity alone, which have been obtained with levofloxacin during clinical trials in CF [6,18].

## 4. Materials and Methods

### 4.1. Bacterial Strains

Nonduplicate clinical isolates of *P. aeruginosa* from a permanent collection for the storage of bacterial strains of unrecognizable patients with CF were grown in Luria broth (LB) (Sigma-Aldrich, Milan, Italy). Minimum inhibitory concentrations (MICs) were determined using the reference broth microdilution methodology according to the Clinical and Laboratory Standards Institute (CLSI) guidelines [19] for levofloxacin, colistin, aztreonam, and amikacin for all isolated strains. EUCAST v.12 clinical breakpoints were used for interpretation of susceptibility data, where available. After 18 h of incubation at 37 °C, the MICs of the aforementioned antibiotics were defined by no visible growth on plates. Since it has been shown that LOX is mainly produced in Pseudomonas strains that produce biofilms, we have preliminarily evaluated the ability of selected strains to produce biofilm [20].

### 4.2. Biofilm Quantification

One microliter of a late-log-phase culture was added to 99 μL LB in a 96-well microtiter plate, incubated for 10 h at 37 °C. Biofilm production was quantified by measuring the absorbance of crystal violet (Sigma-Aldrich-, Italy). Biofilms were fixed by heat at 60 °C for about 1 h for the crystal violet assay. Subsequently, wells with 150 μL of crystal violet solution (2.3% prepared in 20% ethanol) were incubated for 15 min at room temperature. Excesses of crystal violet were removed by washing and then 200 μL of 33% glacial acetic acid was used to dissolve dye fixed to the biofilm and it was incubated for 1 h at room temperature. CV absorbance was measured at 570 nm using a microplate spectrophotometer (BioTek™ Epoch 2 Microplate Spectrophotometer; BioTek Instruments, Winooski, VT, USA). Biofilm formation was evaluated at 12, 24, and 48 h.

### 4.3. Antibiotics

To evaluate the effect of antibiotics, we decided to select three antibiotics with different mechanisms of action. Aztreonam inhibits bacterial cell wall formation, colistin interferes with membrane phospholipids, and levofloxacin inhibits protein synthesis. Aztreonam, colistin, and levofloxacin were purchased from the market (colistin sulfate, aztreonam and levofloxacin for microbiological assay, European Pharmacopoeia (EP) Reference Standard, Sigma Aldrich S.r.l., St. Louis, MO, USA). Antibiotic activity was evaluated against LOX activity and lipid production in a medium containing antibiotics at increasing concentrations (0.5- to 8-fold the MICs in broth).

### 4.4. Lipoxygenase Assay

*P. aeruginosa* strains were grown as single colonies on LB agar overnight. Then, a single colony was inoculated in 10 mL of LB broth and grown to stationary phase under static conditions at 28 °C. Subsequently the culture broth was centrifuged at 8000× *g* for 15 min and the supernatant was collected in a syringe, sterilized, and stored at −80 °C. 10 mL of sample (concentrated supernatants) was mixed with 100 mL of solution A (0.5 mM purified lipoxygenase assay, 10 mM (dimethyl-amino)-benzoic acid (DMAB) (Sigma-Aldrich-Italy) prepared in 100 mM phosphate buffer (pH 6) and incubated for 20 min before the addition of 100 mL of supernatant from each well was transferred into a new plate, and their absorbance was measured at 598 nm.

### 4.5. Culture and Infection of Pulmonary Epithelial Cells

Human pulmonary epithelial NCI-H929 cells were obtained from the American Type Culture Collection (ATCC, Manassas, VA, USA). Cells were grown in RPMI 1640 Glutamax (Gibco, Life Technologies, Rodano, Italy) medium supplemented with 10% heat-inactivated Fetal Bovine Serum (FBS) (Sigma-Aldrich-Italy) in a humidified incubator with 5% CO_2_ at 37 C. For infection experiments, cells were cultivated in 24-well plates until confluence (5.105 cells per well). Exponential growth phase bacteria (LB, 37C, 180 rpm, OD 600 nm) were washed twice in ice-cold PBS before addition to freshly dispensed cell culture medium to obtain MOI = 0.1. After 20 h, supernatants were then centrifuged at 8000× *g* for 10 min, collected in a syringe, and sterilized with a 0.22 mm polymer filter before immediate snap-freezing and storage in liquid nitrogen until lipid mediator extraction.

### 4.6. Lipid Extraction and Liquid Chromatography/Tandem Mass Spectrometry (LC-MS/MS)

We then evaluated the 15-LOX-dependent metabolites, 15-hydroxy-octadecadienoic acid (15-HETE) and 17-HDoHE, and lipoxin A4 (LXA4) in cell lysates and supernatants. Solid phase extraction was performed with HRX-50 mg 96-well plates. Simultaneous separation of the lipids of interest was performed as well as LC-MS/MS analysis on an ultra-high-performance liquid chromatography system (Q Exactive™ Plus Hybrid Quadrupole-Orbitrap™ Mass Spectrometer Thermo Scientific™, Waltham, MA, USA).

### 4.7. Statistical Methods

Continuous variables are expressed as mean and standard deviations (sd). To compare the effects on LOX, 15-HETE, 17-HDoHE, and LOXa4 activity according to the different antibiotics at increasing concentrations, we used a two-way analysis of variance (ANOVA) followed by post hoc comparison using Bonferroni *t*-test. *p*-values of <0.05 were considered significant.

Statistical analyses were performed using GraphPad InStat 8 (GraphPad Software Inc., La Jolla, CA, USA).

## 5. Conclusions

Among clinically isolated strains of *P. aeruginosa* from patients with CF, we selected the strain with highest activity of biofilm production and LOX activity.

Testing levofloxacin, aztreonam, colistin, and amikacin at increasing concentrations, only levofloxacin showed a significant activity on the secretion of LOX. Levofloxacin also showed a significant activity on 15-HETE, 17-HDoHE, and LXA4 production. In conclusion, levofloxacin is the only antibiotic, among those studied according to the mechanism of action and the approval for aerosol use, which can impact the secretion of LOX in a strain with a high ability of biofilm production.

## Figures and Tables

**Table 1:** MICs of levofloxacin, amikacin, aztreonam and colistin against *P. aeruginosa strains isolated from patients with cystic fibrosis*.

	Levofloxacin	Amikacin	Aztreonam	Colistin
**Strain 1**	8	8	<0.25	>64
**Strain 2**	4	1	<0.25	8
**Strain 3**	4	2	<0.25	0.5
**Strain 4**	<0.25	<0.25	<0.25	<0.25
**Strain 5**	<0.25	1	4	0.5
**Strain 6**	<0.25	1	4	0.5
**Strain 7**	0.25	1	4	1
**Strain 8**	0.25	<0.25	<0.25	<0.25
**Strain 9**	4	1	16	<0.25
**Strain 10**	8	4	32	<0.25
**Strain 11**	8	8	8	16
**Strain 12**	16	32	8	2

**Table 2:** One-way ANOVA effects of different antibiotics on LOX activity.

Bonferroni’s Multiple Comparison Test	Mean Difference	t	*p* < 0.05	95% CI
Levofloxacin vs. aztreonam	−0.7200	4.237	Yes	−1.273 to −0.1673
Levofloxacin vs. amikacin	−0.4800	2.824	No	−1.033 to 0.07266
Levofloxacin vs. colistin	−0.6680	3.931	Yes	−1.221 to −0.1153
Levofloxacin vs. control	−1.180	6.943	Yes	−1.733 to −0.6273
Aztreonam vs. amikacin	0.2400	1.412	No	−0.3127 to 0.7927
Aztreonam vs. colistin	0.05200	0.3060	No	−0.5007 to 0.6047
Aztreonam vs. control	−0.4600	2.707	No	−1.013 to 0.09266
Amikacin vs. colistin	−0.1880	1.106	No	−0.7407 to 0.3647
Amikacin vs. control	−0.7000	4.119	Yes	−1.253 to −0.1473
Colistin vs. control	−0.5120	3.013	No	−1.065 to 0.04066

## Data Availability

Data available on request from the authors upon reasonable request.
